# Trigeminal Nerve Schwannoma: A Rare Case of Multisymptomatic Cranial Nerve Involvement

**DOI:** 10.7759/cureus.74919

**Published:** 2024-12-01

**Authors:** Inês S Pinheiro, Carolina Morgado, Ana Margarida Novo, Laura Baptista, Ana Rita Barbosa

**Affiliations:** 1 Internal Medicine, Unidade Local de Saúde da Região de Aveiro, Aveiro, PRT; 2 Neurology, Laboratório de Neuropatologia, Unidade Local de Saúde de Coimbra, Coimbra, PRT

**Keywords:** gait imbalance, mesencephalic-pontine lesion, neurological examination, nystagmus, schwannoma, trigeminal nerve

## Abstract

Schwannomas (SCs) are benign tumors composed of neoplastic Schwann cells and are relatively uncommon intracranially. Although these tumors are frequently associated with neurofibromatosis type 2 (NF2), they may also arise idiopathically, and their pathogenesis remains poorly understood. A 70-year-old Caucasian man presented with a two-month history of vertigo, gait imbalance, and decreased visual acuity in the left eye accompanied by photophobia, nausea, vomiting, and occasional headaches. On physical examination, he exhibited hyposmia, horizontal nystagmus, superior oblique palsy, decreased photoreactivity of the left pupil, hypoesthesia in the middle and lower left facial regions, and a positive Romberg sign. Non-contrast computed tomography (CT) revealed a hypodense, expansive lesion in the left mesencephalic-pontine region. Based on the radiological characteristics, the radiologist suggested ischemic injury or neoplasm as the leading diagnostic hypothesis. Magnetic resonance imaging (MRI) with contrast revealed a cystic-necrotic lesion with multiloculated characteristics in the left mesencephalic-pontine area, with significant mass effect and compression of the brainstem and adjacent ventricular pathway. The lesion was suspected to be a schwannoma, and the patient underwent surgical resection via left temporal and suboccipital craniotomy. Histopathological examination confirmed a schwannoma of the left trigeminal nerve. SCs are most commonly diagnosed in the second and third decades of life, often growing slowly and remaining asymptomatic until they reach a size sufficient to cause functional impairment. MRI is the imaging modality of choice, but histologic confirmation remains the gold standard for diagnosis. This case underscores the rarity of trigeminal nerve SCs presenting with symptoms resulting not only from the affected nerve but also from compression of adjacent structures, such as the vestibulocochlear and abducens nerves. It also highlights the importance of maintaining a high index of suspicion when diagnosing rare intracranial SCs. Advanced imaging techniques and comprehensive clinical evaluation are crucial for identifying complex neurological conditions, particularly when initial findings, such as CT results, suggest alternative diagnoses like stroke.

## Introduction

Schwannomas (SCs) are benign tumors composed of neoplastic Schwann cells, which do not commonly occur intracranially. Intracranial SCs account for approximately 8% of all primary brain tumors, and intraparenchymal SCs are extremely rare [1,2]. Although these tumors are frequently associated with neurofibromatosis type 2 (NF2), they may also arise idiopathically, and their pathogenesis remains unclear [3].

SCs can develop along the course of any peripheral nerve sheath or cranial nerve (CN). Although the most commonly affected CN is the vestibulocochlear nerve (CN VIII), SCs can also occur in the trigeminal (CN V), glossopharyngeal (CN IX), and hypoglossal (CN XII) nerves. SCs involving the oculomotor (CN III), trochlear (CN IV), or abducens (CN VI) nerves are exceedingly rare in the absence of NF2 [2,4]. Characteristic features of these tumors include calcification (29%), perilesional edema (87.5%), and cyst formation (20%). One or two of these findings are present in 70-100% of intraparenchymal SCs, although they are not specific to them [1]. Microscopically, SCs are well-demarcated, encapsulated, and consist of regions with fascicles of spindle-shaped Schwann cells (Antoni A pattern). These areas may transition gradually or abruptly into more loosely organized, microcystic regions (Antoni B pattern) [5]. Due to their rarity, standardized treatment guidelines for intracranial SCs have not yet been established [6,7].

Intracranial tumors can present with a sudden onset of stroke-like symptoms, which may occasionally be misdiagnosed as ischemic stroke. Studies have indicated that 3-5% of brain tumors are initially misdiagnosed as strokes [8,9].

Herein, we report a case of a patient with neurological symptoms, in which the CT scan suggested the possibility of either ischemic injury or neoplasia. Based on the clinical history and progression, we considered a brain tumor or another space-occupying lesion as the main diagnostic hypothesis.

## Case presentation

A 70-year-old Caucasian man presented to the emergency room with a two-month history of vertigo and gait imbalance. The patient reported decreased visual acuity in the left eye with photophobia, which he initially attributed to worsening cataracts. Additionally, he reported nausea, intermittent vomiting, and occasional headaches. His wife noted periods of confusion that began one month earlier. He had a history of type 2 diabetes, essential hypertension, dyslipidemia, cataracts in the left eye (awaiting surgery), and degenerative osteoarticular disease.

On physical examination, the patient presented with hyposmia, horizontal nystagmus, superior oblique palsy, decreased photoreactivity of the left pupil, hypoesthesia in the middle and lower regions of the left side of the face, and a positive Romberg sign.

**Figure 1:**
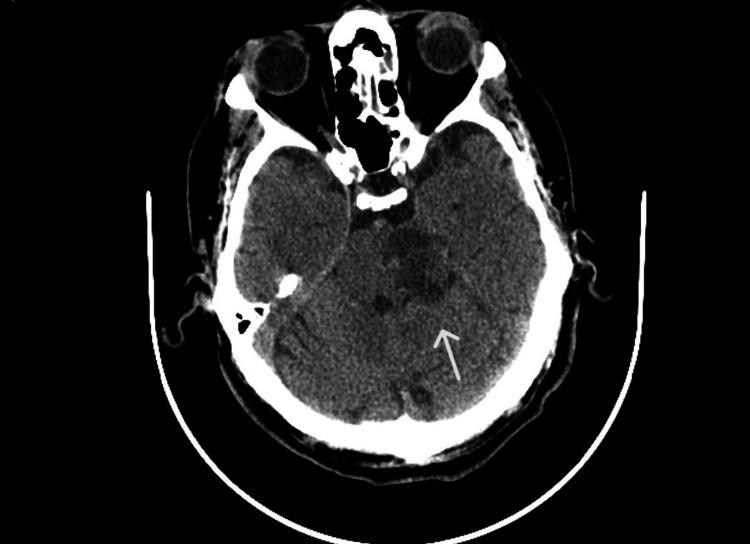
Brain CT scan showing a hypodense lesion with expansile characteristics in the left mesencephalic-pontine region. CT: computed tomography.

A brain computed tomography (CT) scan without contrast was performed, revealing a hypodense lesion with expansive characteristics in the left mesencephalic-pontine region, accompanied by a hyperdense focus of unclear nature (Figure 1). The neuroradiologist proposed the following diagnostic hypothesis: recent ischemic lesion or neoplasm. The patient was subsequently admitted for further evaluation by the internal medicine team.

Blood analyses indicated that vascular risk factors were well controlled (LDL 67 mg/dL, HDL 51 mg/dL, HbA1c 6.1%), and cervical Doppler ultrasound did not reveal significant stenosis in the cervical arteries.

**Figure 2:**
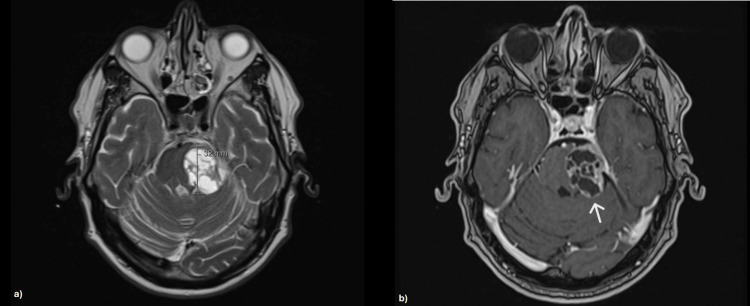
Brain MRI showing an expansive lesion in the left mesencephalic-pontine area (axial view). (a) Proton density + T2 sequence highlighting a globular shape with regular, well-defined borders, measuring approximately 32 mm (anteroposterior). (b) T1 sequence displaying cystic-necrotic/multiloculated characteristics. MRI: magnetic resonance imaging.

**Figure 3:**
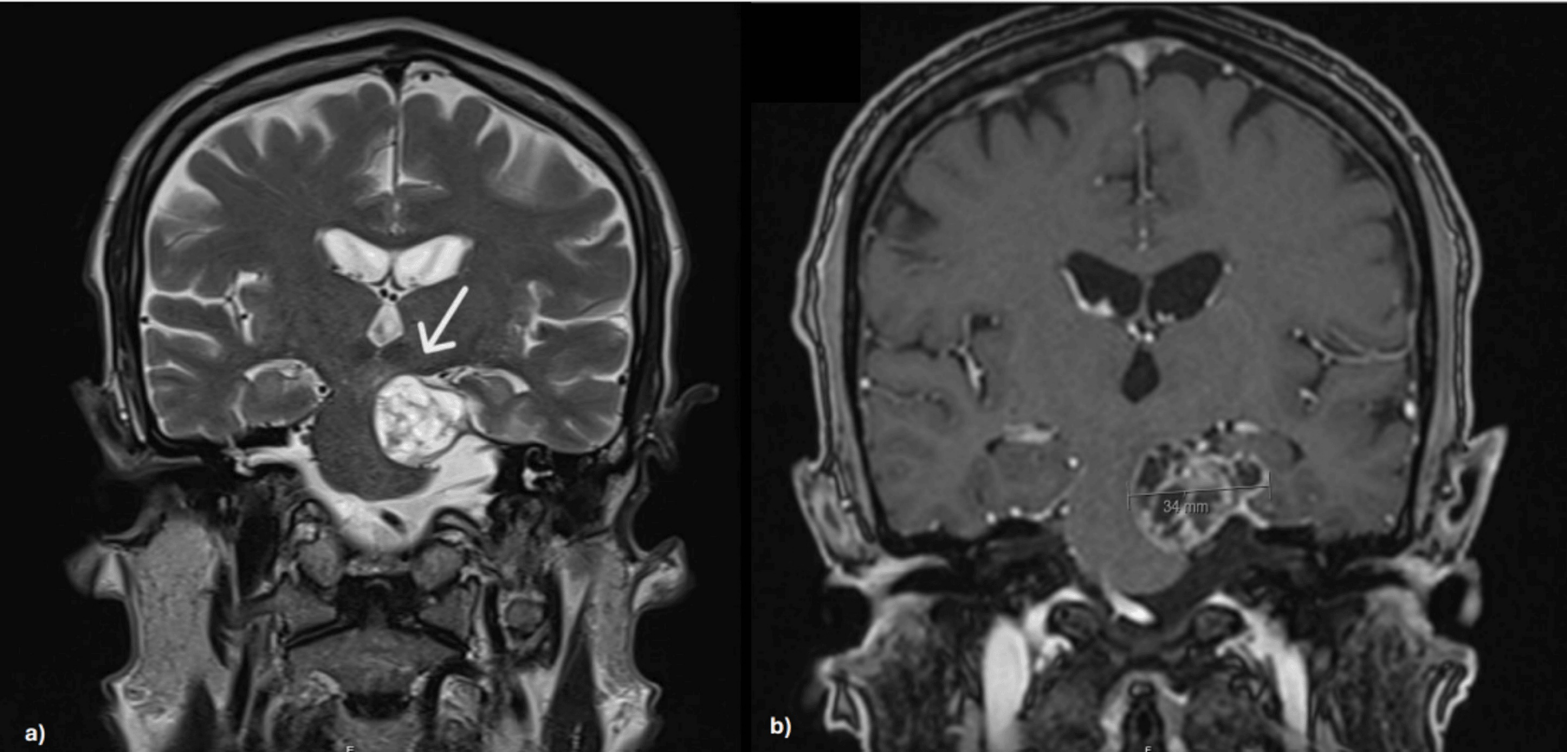
Brain MRI showing an expansive lesion in the left mesencephalic-pontine area (coronal view). (a) Proton density + T2 sequence highlighting a globular shape with regular, well-defined borders. (b) T1 sequence displaying cystic-necrotic/multiloculated characteristics, measuring approximately 34 mm (coronal). MRI: magnetic resonance imaging.

**Figure 4:**
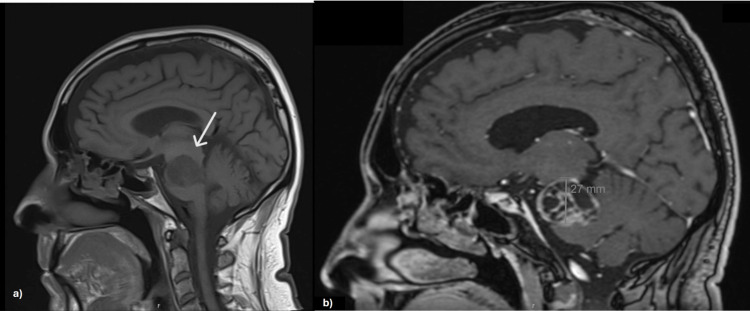
Brain MRI showing an expansive lesion in the left mesencephalic-pontine area (sagittal view). (a) T1 sequence showing compression of brainstem and adjacent ventricular pathway; (b) T1 sequence displaying cystic-necrotic/multiloculated characteristics, measuring approximately 27 mm (vertical). MRI: magnetic resonance imaging.

Further evaluation with contrast-enhanced brain MRI demonstrated a T1-weighted expansive lesion with cystic-necrotic/multiloculated characteristics in the extra-axial region of the left mesencephalic-pontine area. The lesion had a globular shape with regular, well-defined borders, measuring approximately 27 mm (vertical-sagittal view) × 34 mm (coronal view) × 32 mm (anteroposterior-axial view) (Figures 2-4). It almost completely obliterated the adjacent cisternal space and was contiguous with the tentorium cerebelli, causing significant mass effect and contralateral displacement of the brainstem, as well as moderate compression of the fourth ventricle. It established cleavage with adjacent structures, altering the topography of the vascular pathways of the posterior circulation and the neural pathways, particularly affecting the IV, V, and VI cranial nerves. The lesion was considered in the differential diagnosis of schwannoma.

The case was discussed with the Neurosurgery department. The patient underwent a left temporal and suboccipital craniotomy, posterior petrosectomy, and tumor resection. The infratentorial and supratentorial lesion was removed in small pieces using an ultrasonic aspirator and bipolar coagulation. Macroscopically, the lesion was consistent with a schwannoma of the fifth CN (trigeminal nerve), compressing adjacent structures.

**Figure 5:**
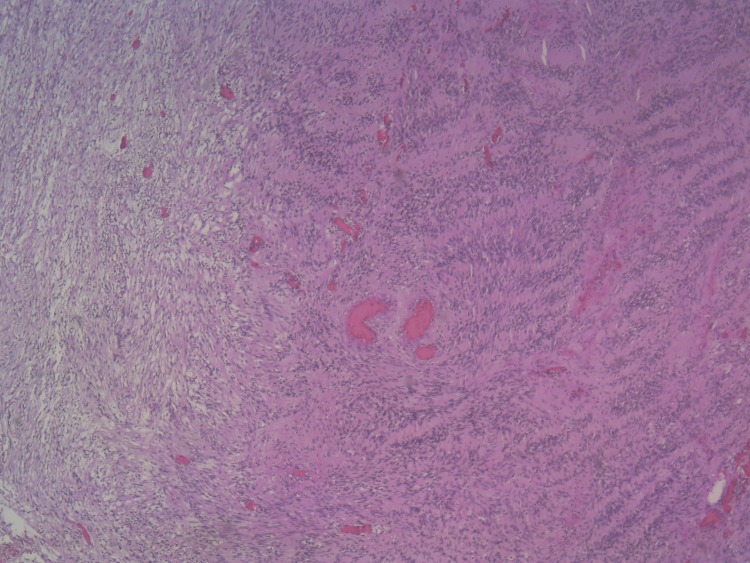
Histopathological result of the tumor. H&E (hematoxylin and eosin stain) 50x: Transition between hypercellular areas of elongated cells arranged in parallel or interlaced bundles (Antoni A areas) and occasionally disposed in nuclear palisades with eosinophilic, anucleated areas between them, forming Verocay bodies. These regions are contrasted with less cellular areas consisting of round nuclei with poorly defined cytoplasm in a microcystic background (Antoni B areas).

The histopathological result revealed findings consistent with schwannoma of the left trigeminal nerve. Hypercellular areas composed of elongated cells arranged in parallel bundles were observed, corresponding to Antoni A areas. Additionally, hypocellular regions were identified, consisting of cells with round nuclei and poorly defined cytoplasm in a microcystic background, characteristic of Antoni B areas (Figure 5).

Postoperatively, the patient recovered well from surgery, with improvement in his neurological deficits. He was hospitalized for 25 days, during which gradual improvement was observed, including the resolution of facial hypoesthesia. At the time of discharge, the patient exhibited residual paresis of the fourth CN (vertical strabismus) and dysfunction of the eighth CN, characterized by imbalance, complete loss of the head impulse reflex, and ipsilateral hearing loss.

## Discussion

SCs are more commonly observed in the second and third decades of life, with both sexes being equally affected. These tumors are typically asymptomatic and exhibit slow growth until they reach a size sufficient to cause functional impairment. Patients with trigeminal nerve SCs often present with symptoms related to nerve dysfunction, with facial pain being the most prevalent complaint [10,11]. In addition to trigeminal nerve involvement, which is often associated with supratentorial SCs, lesions involving the vestibulocochlear nerve (CN VIII) and the olfactory nerve (CN I) have also been described in this location [1]. MRI is the imaging modality of choice due to its multiplanar capabilities and superior soft tissue contrast, making it invaluable for both diagnosis and surgical planning. However, its definitive diagnosis is achieved through histopathological result [11,12]. 

When completely excised, sporadic SCs typically do not recur, and the prognosis is generally excellent. Malignant transformation is extremely rare, and radiotherapy is not part of the standard treatment protocol, as these tumors are inherently radioresistant [11].

Here, we present a rare trigeminal nerve schwannoma, with symptoms arising not only from the involvement of this nerve but also from the compression of adjacent structures.

This case highlights several noteworthy aspects. First, it underscores the critical importance of a thorough neurological examination in identifying CN involvement. The patient initially attributed his symptoms to cataracts (including photophobia and headaches), underestimating the severity of his condition and delaying the recognition of his neurological deficits. Additionally, the duration of symptom progression provided valuable diagnostic insights, aiding in the differentiation between an acute event and a more insidious pathological process. In this instance, the patient reported symptoms persisting for at least two months, prompting clinicians to consider differential diagnoses beyond a recent ischemic event, as initially suggested by the CT scan findings.

This case also emphasizes the utility of MRI as a high-sensitivity imaging modality, which proved indispensable in refining the diagnosis when CT results were inconclusive. The superior soft tissue contrast and multiplanar capabilities of MRI facilitated a more detailed assessment, revealing the complex cystic and necrotic characteristics of the lesion.

## Conclusions

The clinical presentation of trigeminal nerve SCs is typically associated with trigeminal nerve dysfunction, such as facial pain, numbness, or paresthesia. However, this case demonstrates that these tumors may also elicit symptoms due to the compression of adjacent structures. The patient exhibited vestibulocochlear and abducens nerve-related symptoms, indicating that the tumor's mass effect extended beyond the trigeminal nerve.
